# Unusual Cystic Variant of Calcifying Epithelial Odontogenic Tumor

**DOI:** 10.30476/DENTJODS.2019.77772.

**Published:** 2020-06

**Authors:** Mala Kamboj, Achla Bharti Yadav, Anjali Narwal, Neera J

**Affiliations:** 1 Dept. of Oral Pathology & Microbiology, Post Graduate Institute of Dental Sciences, Rohtak, Haryana, India;; 2 Dept. of Oral Pathology, D J College of Dental Sciences & Research, Modinagar, India;; 3 Dept. of Dentistry, GMCH 32, Chandigarh, India.

**Keywords:** Calcifying epithelial odontogenic tumor, Cystic variant, Maxilla, Odontogenic

## Abstract

Calcifying epithelial odontogenic tumor (CEOT) is a rare benign odontogenic neoplasm, which is exclusively epithelial in its tissue of origin. Many cases of CEOTs are associated with impacted tooth and simulate dentigerous cyst radiographically. The histologic features of CEOT are unique; however, among its various histologic subtypes, the cystic variant is a rare and less well-understood entity. Our report elucidates a cystic variant of CEOT in the maxilla of a 16-year-old male that presents clinical and radiologic findings conscientious to dentigerous cyst; but histopathological diagnosis came out to be a gold standard in identifying this rare tumor. This case report describes the clinicopathologic features of this rare entity, highlighting the histomorphological findings along with reviewing other reported cases.

## Introduction

Foremost, calcifying epithelial odontogenic tumor (CEOT) was introduced by Dr. Jens J Pindborg in 1956, following which Pindborg tumor eponym was given in 1967. In 1992, World Health Organization (WHO) has classified it as a benign odontogenic neoplasm, which is utterly epithelial in origin [ 1 ]. CEOT is a rare entity relatively accounting 1% of all odontogenic tumors. Classically, it is a benign, slow growing but locally aggressive neoplasm and it tends to invade bone and adjacent soft tissue [ 2 - 3 ]. Along with epithelium rich, amyloid/ calcification rich and balanced distribution of epithelium and amyloid in CEOT other histological presentations have also been described (non- calcifying/ clear cell/ cystic/ cribriform) in the literature [ 4 ]. Still, exact typing of CEOT is not done which can be beneficial for surgeons to determine better treatment plan and prognosis. The cystic variant of CEOT is the rare type and six cases of this variant have been documented in the literature until this report [ 2 - 5 ]. The present case report is about cystic variant of CEOT emerging in the maxilla along with review of other reported cases. 

## Case Report

A 16-year-old male patient reported to the Dental Department of our Institute for the complaint of swelling and pain on left side of face, which had developed 2-3 months earlier. The swelling occurred insidiously and enlarged gradually to the present size over the past 2 months. There was associated pain but paresthesia was absent in the region.

**Figure 1:**
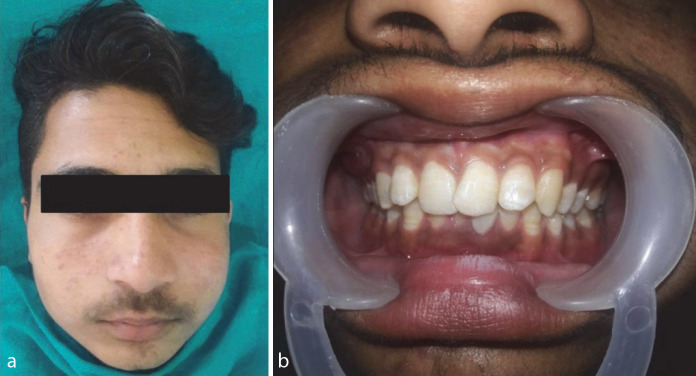
a; Clinical extra- oral and b; intra- oral view

Clinically, a diffuse swelling was present in the middle third of the face measuring approximately 3.5cm x 3.5cm, extending superio- inferiorly from below left infraorbital rim to left lip commissure and medio- laterally from left ala of nose to 3-4cm in front of left tragus. Intra oral examination revealed a diffuse swelling obliterating the buccal vestibule in relation to the left maxillary premolar- molar region (Figure 1).

**Figure 2:**
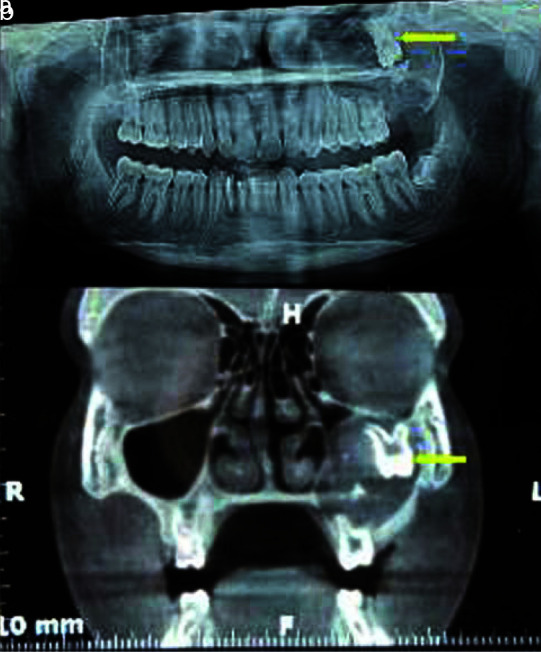
**a:** Panoramic radiography revealing unilocular radiolucency above the root apices of left maxillary second premolar and first molar involving unerupted left maxillary second molar in center, **b:** Computed tomography (CT) scan shows a well- defined unilocular radiolucency in the left maxillary sinus, with impacted tooth and few flecks of calcified deposits

**Figure 3:**
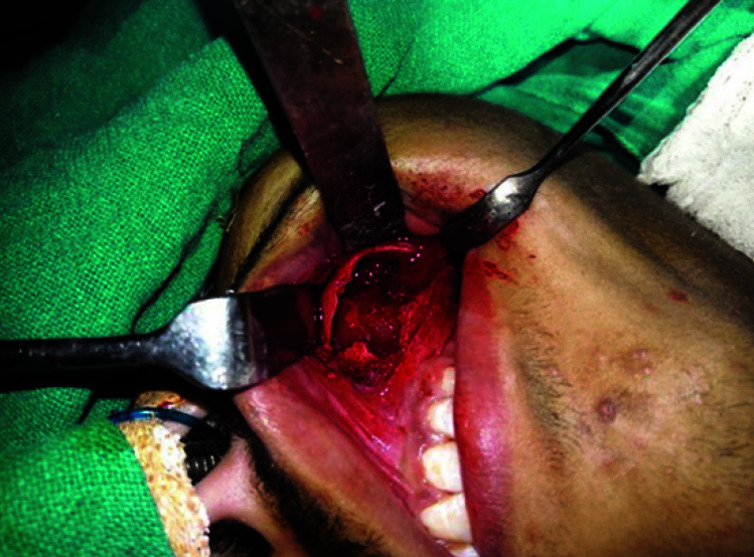
Intra- operative picture of surgical approach

**Figure 4:**
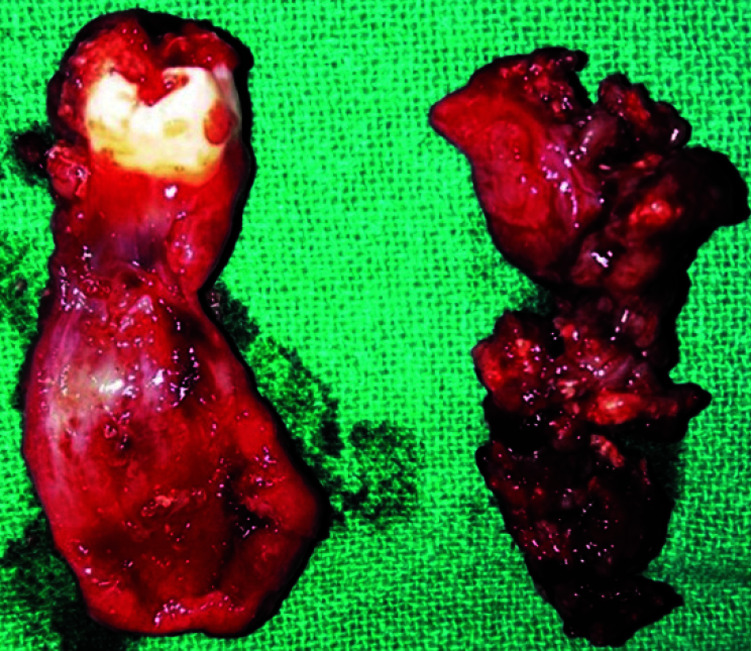
one large cystic specimen attached at the cemento- enamel junction of the impacted tooth and other separated bit

On palpation, swelling was firm, non- compressible, non- fluctuant and afebrile in nature. On radiographic examination, dental panoramic imaging revealed a relatively homogenous radiolucency above the root apices of left maxillary second premolar and first molar, pushing the lateral wall of maxillary sinus, extending up to the orbital floor, involving unerupted left maxillary second molar in center with dubious radiopaque content. Computed tomography (CT) showed a well- defined unilocular radiolucency in the left maxillary sinus, with impacted tooth and few flecks of calcified deposits. The lesion expanded the maxilla, eroded the lateral sinus wall, and projected into the nasal cavity (Figure 2). A provisional radiographic diagnosis of dentigerous cyst or CEOT was given. Fine needle aspiration was uneventful; therefore, the lesion was enucleated with all the clear margins (Figure 3). Gross examination revealed two irregular fragments, one large cystic specimen with lining attached at the cemento- enamel junction of the impacted maxillary left second molar. The other bit was separated from the lumen of the cyst (Figure 4). Microscopically, odontogenic epithelial lining of varied thickness was reported at the cystic portion of the lesion. The majority of the lining shows 12-15 cell layer thickening characterized by polyhedral cells with abundant eosinophilic cytoplasm and centrally placed nuclei. 

**Figure 5:**
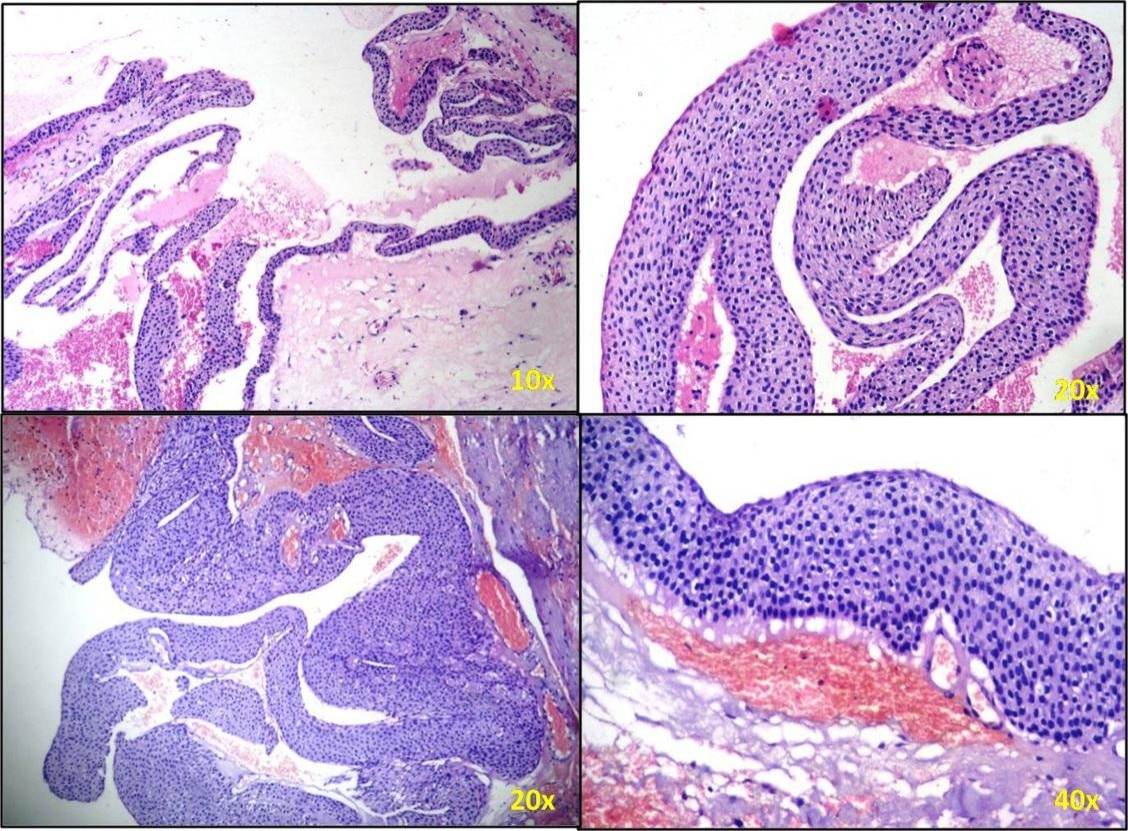
Cystic cavity lined by odontogenic epithelium of varying thickness composed of polyhedral cells with a distinct outline and centrally placed hyperchromatic nuclei (H&E; 10x, 20x, 40x)

**Figure 6:**
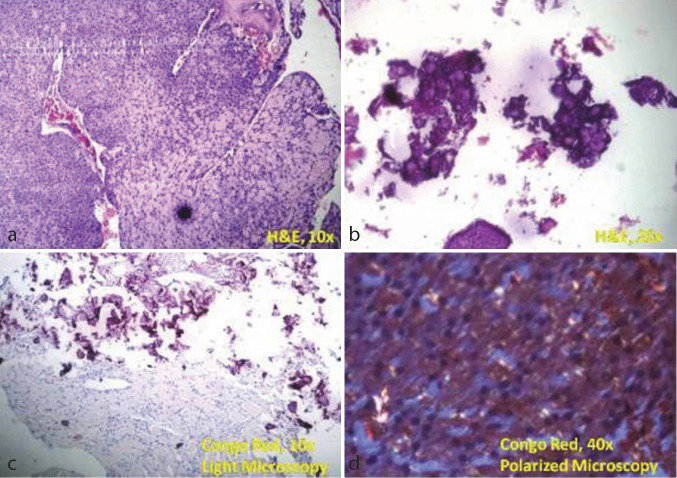
Sheet of polyhedral epithelial cells interspersed by calcifications and homogeneous eosinophilic amyloid-like material stained positive for Congo red and exhibiting minimal apple-green birefringence under polarizing microscope (H&E; 10x, 20x and Congo red;10x, 40x).

Surrounding capsule shows features of hyalinization and separation of epithelial and connective tissue in few areas (Figure 5). The other bit that was processed in a separate tissue block revealed sheets of tumor cells with features typical of CEOT like polyhedral epithelial cells with distinct outlines, abundant cytoplasm, and centrally placed hyperchromatic nuclei, areas of amorphous, eosinophilic, hyalinized material that was positive for Congo red stain and manifesting minimal apple-green birefringence under polarizing microscope. Multiple calcifications were also present (Figure 6). Considering all the prominent features, the final diagnosis of cystic variant of CEOT was made. The patient was followed up for six months with no sign of clinical recurrence. Informed consent was obtained from the patient for publishing her clinical photography and radiography. 

## Discussion

CEOT is classified as an uncommon, benign, odontogenic neoplasm that is exclusively epithelial in its tissue of origin, incidence of which varies between 0.4% and 3% of all odontogenic tumors [ 3 , 5 ].

The tumor prevailed over a broad range of age, most commonly between 30 to 50 years of age with mean of 40 years. It does not have a racial or sex predilection [ 2 - 3 ]. Between two illustrious topographic variants, the majorities (approx. 94%) are of intraosseous (central/ intrabony) type arising predominantly at the mandible and a rare (approx. 6%) extraosseous (peripheral/ soft tissue) type commonly occurs at the gingiva. The central type presents 2 to 1 ratio of mandible to maxilla with most common location in the premolar and molar regions. Radiologically, intraosseous variant manifest radiolucent areas with varied amount of calcifications, while the extraosseous type shows superficial bone erosion [ 6 - 7 ]. Almost half of the cases are associated with unerupted/ impacted teeth and appear radiographically as dentigerous cysts [ 2 ]. Histogenesis of CEOT is still uncertain; the intraosseous type presumably originates from the stratum intermedium of enamel and the extraosseous variant apparently derived from dental lamina or the basal cells of gingival epithelium [ 7 ]. 

Microscopically, sheets, islands, and cords of polyhedral eosinophilic epithelial cells with prominent intercellular bridges, some degree of nuclear pleomorphism, hyperchromatism, and prominent nucleoli, characterize the CEOT. Homogeneous, eosinophilic, acellular matter intermixed with the tumor cells have been identified as ‘amyloid-like' substance. The inherent characteristics of the amyloid- like material are still inconclusive. Spherical amorphous calcifications may be present in between tumor cells and connective tissue [ 1 - 3 ]. Ai-Ru *et al*. [ 8 ] suggested a classification for CEOT depending on epithelial cytology and histomorphology, stromal density and composition and amyloid/calcification pattern- epithelium rich, balanced distribution, amyloid/ calcification rich. These histomorphological patterns are not c-ommonly used in microscopic description of CEOT [ 4 ]. Several histologic variants of CEOT have been reported, such as clear cell, Langerhans cell containing, cementum forming, non-calcifying, associated or combined with other odontogenic lesions such as adenomatoid odontogenic tumor, with associated cystic lining, and CEOT with myoepithelial cells [ 4 , 9 - 10 ]. 

Until now, six cases were reported in the literature with a true cystic variant of CEOT [ 2 - 5 ]. Clinical, radiological, and histopathological features of the reported cases along with the present case have been summarized in Table 1. Gopalakrishnan *et al*. [ 2 ] and Channappa *et al*. [ 3 ] reported cystic lesion associated with the impacted tooth like the present case but in the case of Barreras *et al*. [ 5 ], impacted tooth was not present. Among all the reported cases only Gopalakrishnan *et al*. [ 2 ] showed area of transition from thin dentigerous cyst like lining into thicker CEOT cystic epithelium, which might contribute to the fact that cystic CEOT arises from the neoplastic transformation of the dentigerous cyst but still debatable.

**Table 1:** Detailed clinical, radiological, and pathological features of individual reported cases of cystic variant of CEOT

Case reported	Age (years)/ Gender	Site	Radiographic presentation	Histopathological findings (H& E)
Gopalakrishnan *et al*.[2]	15/M	Left Maxilla	Unilocular radiolucency in the left maxillary sinus, with calcified deposits surrounding the crown of the impacted tooth (#27)	Cyst lined by odontogenic epithelium that varied in thickness with majority of the lining showed classic features of CEOT Area of transition from thin dentigerous cyst like lining into thicker CEOT cystic epithelium
Channappa *et al*. [3]	30/M	Left Maxilla	Unilocular mixed-density lesion with the presence of a fluid component in the left maxillary sinus region along with calcifications and associated impacted tooth (#25)	Cyst lined by odontogenic epithelium of uniform thickness, with classic features of CEOT
Azevedo *et al*. [4]	In an immunohistochemical study on CEOT, 3 out of 19 cases showing cystic variant of CEOT histologically (Individual clinical & radiological detail of these cases were not separated out by the author)			
Barreras *et al*. [5]	31/M	Left Mandible	Unilocular mixed radiopaque/lucent expansile area eroding the cortex without presence of impacted tooth	Cystic portion featured odontogenic epithelial lining of uniform thickness characterized by cells with optically clear cytoplasm and centrally located round nucleus and presence of calcified material with characteristics of osteodentin. Diagnosis of clear cell cystic variant of CEOT was made
Present case	16/M	Left Maxilla	Homogenous radiolucency above the root apices of left maxillary second premolar and first molar, along the lateral wall of maxillary sinus, involving impacted tooth in centre (#27)	Cystic portion is lined by odontogenic epithelial lining of varied thickness with majority showed classic features of CEOT and areas of eosinophilic amyloid like material and multiple calcifications

Intriguing element in our case was areas of amorphous, eosinophilic, hyalinized material that stained positive for Congo red and exhibited minimal apple-green birefringence under polarizing microscope. Vickers *et al*. [ 11 ] first recognized the presence of amyloid-like material in CEOT, but controversy exists regarding its nature. Earlier, the school of thought was cytokeratins, enamel-related proteins, and basement membrane components could be the possible contents of amyloid-like material. Recently, Solomon *et al*. [ 12 ] designated this unique material as A Pin and concluded that it is a novel protein identical to the N-terminal portion of a 153 amino acid sequence protein; but the biological and clinical significance of A Pin is still unknown.

Franklin and Pindborg suggested that CEOTs are less aggressive with only 14% recurrence rate [ 13 ]. Conventional CEOTs have been universally treated by conservative surgical resections along with removal of a narrow rim of bone. However, the treatment of CEOT is precisely guided by other factors such as site, size, and utmost important histomorphological features of the lesion [ 2 - 3 ]. More number of similar reported cases can illustrate the exact treatment plan and prognosis of cystic CEOTs better. 

This variant should be included in the differential diagnosis for any jaw lesion manifesting as a cyst. Patient’s consent was taken for the publication of this case and he was satisfied with the presentation and treatment.

## Conclusion

Being aware of diversified variants and types of CEOTs, need of the hour is to introduce histological sub-typing of CEOT along with establish criterion for the same. It will further highlight the biological, clinical implications and will be beneficial for the treatment and prognosis of such varied cases. 
